# Changes in biofield measures and experienced states during meditation and breathwork practices: an uncontrolled feasibility study

**DOI:** 10.3389/fpsyg.2026.1623301

**Published:** 2026-02-25

**Authors:** Natalie L. Dyer, Meredith L. Sprengel, Ivo Stuldreher, Koen van der Sanden, Anne-Marie Brouwer, Sébastien Velut, John A. Ives, Yu Yan, Eduard van Wijk

**Affiliations:** 1 Connor Whole Health University Hospitals, Cleveland, OH, United States; 2 Center for Reiki Research, Southfield, MI, United States; 3 Cognitive Psychology Unit, Institute of Psychology, Leiden University, Leiden, Netherlands; 4 Human Factors, Netherlands Organisation for Applied Scientific Research (TNO), Soesterberg, Netherlands; 5 Federation ENAC ISAE-SUPAERO ONERA, Universite de Toulouse, Toulouse, France; 6 A&O—Laboratoire Interdisciplinaire des Sciences du Numérique, Universite Paris, Paris, France; 7 Consultant, Alexandria, VA, United States; 8 Meluna Research, Wageningen, Netherlands

**Keywords:** biofield, breathwork, heart rate, infrared, meditation, ultraweak photon emission

## Abstract

**Background:**

The human body radiates multiple biological fields which can be measured with sensors directly on or off the body. These electromagnetic fields are hypothesized to be altered by mind and body practices. The primary goal of the current study was to evaluate the feasibility of continuous multi-sensor monitoring during meditation and breathwork practices. It also explored methods to investigate whether simultaneous biological field measures correlate as parts of a dynamic system of biofields responsive to changes in states of consciousness.

**Methods:**

Twenty-three adults were recruited to participate. The intervention consisted of a guided loving kindness meditation followed by a guided breathwork exercise while equipped with multiple biofield sensors to measure heart rate (HR), heart rate variability (HRV), skin conductance (SCR), alpha waves with electroencephalography (EEG), infrared radiation (IR), and ultraweak photon emission (UPE). Participants completed self-report measures of emotional affect and states of consciousness. Aggregate and individual differences in participants’ responses to the meditation and breathwork were assessed. Exploratory analyses of within-subject correlations between biofields were also conducted.

**Results:**

The study procedure was feasible with 100% recruitment and retention and the intervention was acceptable to participants. Meditation significantly increased IR of the nose (*p* = 0.003). Breathwork significantly increased HR (*p* = 0.002) and decreased IR of the nose (*p* < 0.001), and left hand UPE showed a near-significant decreasing trend (*p* = 0.057). Biofield measures changed as expected for some participants. Post-meditation, participants reported lower arousal and increased control, boundarylessness, and non-duality. Post-breathwork, participants reported increased arousal and decreased boundarylessness, connectedness, and non-duality. There were strong correlations (r > 0.5) between UPE from both hands and moderate correlations (r > 0.4) between IR nose temperature and left hand UPE.

**Conclusion:**

The current study demonstrated the feasibility of simultaneous measurement using multiple on- and off-body biological field sensors. Meditation and breathwork produced nearly opposite effects on self-report and biofield measures. Preliminary analysis indicated intra-subject correlations between different biofield measures. Future work with a larger sample size and appropriate control groups is required to draw conclusions about the systemic nature of biofield measures and their relationship with states of consciousness.

## Introduction

The human body produces several types of electric currents, potentials and electromagnetic fields (EMFs) which reflect physiological states (Hammerschlag et al., 2015). Bioelectric fields, generated by electrical activity in the brain, heart, and other muscles, are detectable using skin-contact electrodes. Electroencephalography (EEG), electrocardiography (ECG), and electromyography (EMG) are among the most well-understood methods used in clinical diagnosis. Bioelectric signaling inside the body also acts to regulate critical processes such as embryogenesis, regeneration, metastatic transformation, and tumorigenesis (Levin, 2014).

Recent technological advancements have enabled the measurement and analysis of electromagnetic biofields at a distance, including infrared radiation (IR) and biophoton emissions, both of which are measured off the body rather than with on-body sensors. Patterns of body surface heat have been linked to emotions, psychophysiological activity, and affective states in social interactions (Cardone et al., 2015; Clay-Warner and Robinson, 2015; Ioannou et al., 2014; Merla, 2014). IR is perceived by humans as heat and can be detected using optical sensors via infrared thermography (IRT). IRT can measure small changes in body surface temperature influenced by factors including metabolism, muscular activity, subcutaneous blood flow, and perspiration (Ioannou et al., 2014), as such, it has been explored as a non-invasive diagnostic tool for various disease conditions (Kesztyüs et al., 2023). Through IRT, changes in breathing can be identified and used to assess emotional and mental states, as well as to detect disorders involving breathing disturbances such as sleep apnea (Pereira et al., 2015; An et al., 2022).

Ultraweak photon emission (UPE) is the spontaneous emission of visible, IR, and ultraviolet light which emanates from all living organisms (Popp et al., 1984), primarily resulting from the production of reactive oxygen species (Ives et al., 2014). Detection of UPE may be useful as a non-invasive diagnostic and research tool (Ives et al., 2014; Du et al., 2023) and several studies suggest both diagnostic and regulatory roles for UPE (Ives et al., 2014; Yang et al., 2015, 2017), for example in intercellular signaling (Van Wijk, 2001), and it has been suggested that such signaling by phase coherent biophotons could explain many regulatory functions (Popp and Chang, 1998). UPE have been measured from a variety of body locations, and a comparative study date examined 29 body sites (Van Wijk et al., 2005). Results showed that UPE from the hands and head were typically higher than from other body locations. Right–left symmetry is commonly found in people, but not dorsal-ventral symmetry. Furthermore, data suggested a common human anatomical UPE pattern, which was first confirmed with a highly sensitive charge-coupled device (CCD) imaging system (Kobayashi, 2003) followed by a systematic quantification by the photomultiplier system (Van Wijk et al., 2006). Previous studies have reported that UPE at the skin surface increases with oxidative stress (e.g., Kobayashi et al., 2009; Ives et al., 2014; van Wijk et al., 2014; Zhao et al., 2016) and recent research has examined the association of UPE with changes in activity of metabolic networks (Burgos et al., 2016, 2017).

As a sensitive dynamic system, it is theorized that changes in mental states or states of consciousness correspond with changes in the organization or intensity of biofields (Rubik, 2002, 2008). As such, mind–body practices that relax, stimulate or alter perceptual awareness of boundaries with others and the world (e.g., sense of unity, nonduality), such as meditation or breathing techniques, could influence the dynamics of biofields. For example, changes in EEG activity have been reported for both novice and experienced meditators (Aftanas and Golocheikine, 2002; Muehsam et al., 2017; Rodriguez-Larios et al., 2021; Steinhubl et al., 2015). Changes with meditation in ECG activity (Steinhubl et al., 2015; Delgado-Pastor et al., 2013), IR radiation (Singh et al., 2018; Wang et al., 2023), and UPE (van Wijk et al., 2005, 2006, 2008; Zapata et al., 2021a, 2021b) have also been reported. It is well known that breathing practices can alter EEG (Sviderskaya and Bykov, 2006; Zaccaro et al., 2018) and ECG activity (Zaccaro et al., 2018; Giraldo et al., 2023), but to our knowledge no research to date has been conducted specifically on breathing exercises and breathwork practices and its associated changes in IR or UPE.

Despite these findings and their implications for health and our understanding of human psychology, biology, and biophysics, no study has attempted to simultaneously measure multiple biofields on and off-body, including UPE and IR, while engaging in mind–body practices. This approach is a necessary first step to understand how these biofields are differentially altered by different practices, as well as how they might influence each other. Therefore, the primary goal of the current study was to evaluate the feasibility of conducting a study with continuous multi-sensor monitoring during meditation and breathwork practices with regular participants. The secondary goal was to evaluate changes in self-report before and after, and biofield measurements before, during, and after those practices. Our primary hypothesis was that it would be feasible to conduct the study, as measured by recruitment, retention, data completeness and acceptability. Our secondary hypothesis was that mind–body practices would alter self-report and biofield measures. Our third hypothesis was that correlations would be observed among the various biofield measures.

## Materials and methods

### Study design

This study was a pre-post pilot study of participants performing a meditation and then a breathwork exercise. This study was part of a larger study in which the participants also underwent additional interventions eliciting different mental and emotions using the same measures.

### Participants and setting

Due to the exploratory nature of the current study, a sample size calculation was not performed, and the sample size was determined by number of participants meeting inclusion criteria (see Appendix) and enrolling within the recruitment window of 1.5 months. Twenty-three adults participated in the study. The mean age of participants was 42.3 (SD = 20) and 61% were female. All participants except for one were Caucasian (96%) (see Table 1).

**Table 1:** Demographics of all participants (*N* = 23).

Demographic variable	*N* (%)
Race/Ethnicity	
Caucasian	22 (95.7)
Asian	1 (4.3)
Gender	
Female	14 (60.9)
Male	9 (39.1)
Age	
18–24	6 (26.1)
25–34	6 (26.1)
35–44	1 (4.3)
45–54	2 (8.7)
55–64	2 (8.7)
65–74	6 (26.1)

The study took place at Netherlands Organisation for Applied Scientific Research (TNO) in Soesterberg, The Netherlands. All participants were recruited from the TNO participant pool or through word of mouth. Participants received a remuneration of $50 for completing the study. All study procedures were approved by the TNO Institutional Review Board.

### Outcome measures

#### Self-report measures

Emotional response was measured using the Self-Assessment Manikin (SAM), a 3-item scale that employs a series of five abstract characters along a 9-point scale to measure three dimensions of emotional response: valence (SAMV), arousal (SAMA), and dominance/control (SAMD; Bradley and Lang, 1994). The SAM has satisfactory validity (internal consistency coefficient ranging between 0.63 and 0.98) (Backs et al., 2005).

Changes in states of consciousness related to perceptions of non-duality and connectedness or boundaries with the world and others was assessed with the Inclusion of Self and Others (IOS), Perceived Body Boundaries Scale (PBBS), Spatial Frame of Reference Continuum (S-FORC), and the Nondual Awareness Dimensional Assessment (NADA).

Inclusion of Self and Others (IOS) is a 1-item visual measure developed to assess how close a participant feels to another person or group. Respondents see seven pairs of circles that.

range from just touching to almost completely overlapping. One circle in each pair is labeled “self,” and the second circle is labeled “other.” Respondents choose one of the seven pairs to answer the question, “Which picture best describes your relationship with [this person/group]?” (Aron et al., 1992).

Perceived Body Boundaries Scale (PBBS) is a 1-item visual measure developed to assess a person’s perceived boundaries. The PBBS was developed to help capture the experience of annihilation unity experienced during mediative practices and has good validity (internal consistency coefficient of 0.88) (Dambrun, 2016).

Spatial Frame of Reference Continuum (S-FoRC) is a 1-item visual measure developed to assess the degree to which an individual feels their field of awareness extends beyond the physical body (Hanley et al., 2020; Hudak et al., 2021). This scale has been used in conjunction with the PBBS to measure the relationship between the expansion of awareness and the dissolution of boundaries during loving kindness mediation.

Nondual Awareness Dimensional Assessment (NADA) State is a 3-item questionnaire designed to only measure state nonduality as characterized by relational unity (oneness), ego dissolution, and bliss. The NADA-State has good validity (internal consistency coefficient of 0.73) (Hanley et al., 2020; Hanley et al., 2018).

#### Biofield measures

##### Electrophysiology: heart rate (HR), heart rate variability (HRV), EEG, and electrodermal activity (EDA)

A Biosemi amplifier, ActiveTwo Mk II system (Biosemi, Amsterdam, Netherlands) was used for recording EEG, ECG and electrodermal activity (EDA) sampled at 512 Hz. EEG was recorded with 32 active Ag/-AgCl electrodes, placed on the scalp according to the 10–20 system, together with a common mode sense active electrode and driven right leg passive electrode for referencing. The electrode impedance threshold was 20 kOhm. For EDA, two passive gelled Nihon Kohden electrodes were placed on the ventral side of the distal phalanges of the middle and index finger. For ECG, two active gelled Ag/-AgCl electrodes were placed at the right clavicle and lowest floating left rib.

##### Infrared (IR)

The source of the infrared data was the infrared video recorded using a FLIR A655sc, which is sensitive radiation with wavelengths from 7.5 to 14 micrometer (long-wave infrared or LWIR), and transforms this data into estimated surface temperatures. The camera records relative temperatures with an accuracy of <30 millikelvin (mK). The time resolution of the camera was 25 Hz but was down sampled during data processing to 12.5 Hz to manage file size. The resolution of the infrared video is 640×480 pixels.

##### Ultraweak photon emission (UPE)

UPE were measured using a custom-built two-hands desktop photon counting system, designed for simultaneous, non-invasive measurements of both hands. The equipment was provided by Meluna Research B.V. (Wageningen, The Netherlands). The system (PMS07) has low background noise (5–10 dark counts per second). The system included two measurement units including a photomultiplier and housing located on top of each dark chamber, two control boxes for measurement control and data acquisition plus one computer with data acquisition and analyzing software. The photomultiplier tubes (PMT) (type 9235QA; ET Enterprises) are sensitive in the spectral range of 160–630 nanometers (nm), had a 51-millimeter (2 in.) diameter window and a peak quantum efficiency of 30%. Measurements were controlled automatically vis-à-vis computer software. Photon counts were measured in 50 millisecond intervals. UPE measurements of the left hand were lost for five participants due to a broken connector cable.

#### Interventions

##### Loving kindness meditation

Loving kindness meditation is a practice based on the Buddhist concept of Metta or loving-kindness, whereby compassion and wishes for well-being are directed toward the self and others. The practice is designed to create changes in emotion, motivation, and behavior to promote positive feelings and kindness toward the self and others, and evoke a boundless warm-hearted feeling (Salzberg, 1995). For the loving-kindness meditation, participants followed a 16-min loving kindness (Metta) audio guided meditation by Tara Brach found here: https://www.tarabrach.com/guided-meditation-loving-this-life-happiness-metta-practice-2/. In brief, the meditation focused on generating an inner smile, spreading that smile throughout the body relaxing each corresponding body part, and cultivating spaciousness and presence.

##### Breathwork practice

The breathwork practice was delivered as a guided video from the Wim Hof Method (WHM). The WHM is a high ventilation breathwork practice derived from both Buddhist Tummo meditation and yogic pranayama breathing. The WHM often involves exposing the human body to cold temperatures (Fincham et al., 2023), which was not done in the current experiment. The video of the breathwork practice that was used for this experiment can be found here: https://www.youtube.com/watch?v=0BNejY1e9ik.

The breathwork practice consisted of three intervals of vigorously breathing followed by holding the breath for increasing lengths of time (30 s, 60 s, and then 90 s). This is a slower paced WHM breathwork exercise that is suitable for beginners. Wim Hof provides the voiceover instructions, which is accompanied by background sounds and music, and a video of a “breathing bubble” with a count down. The breathing bubble is an audiovisual guide that helps maintain rhythm and pace during the breathing sessions. The bubble expands and contracts, and the individual follows with their breath.

#### Overall set-up and data collection

Each participant arrived and checked in to the TNO Soesterberg facility in Soesterberg, Netherlands and the inclusion and exclusion criteria were reviewed (see Appendix). If participants were eligible, informed consent was obtained. Participants were then asked to put on gloves to shield their hands from ambient light which was necessary for an accurate reading of the biophoton emissions. The participants then completed self-report questionnaires including demographics and other questionnaires that were not included in the current study, for personality, resilience, and quality of life. The participants were informed that it is important to remain relatively still during the experiment and that there would be short breaks during which they could talk with the experimenter and relax between the different parts of the experiment. The participants were also made aware that the experimenters would remain with them in the room at all times and would be available if they experienced any discomfort or had any questions about the experiment.

Participants were then fitted with the biofield measuring equipment: (1) BioSemi ActiveTwo MkII- physiological data collection using a 32 electrode EEG cap fitted with 32 gelled electrodes; electrodes on the hands to record electrodermal activity; electrodes on the chest to record heart rate and heart rate variability. (2) Photomultiplier tube hand boxes- UPE was taken from the hands using light-tight boxes which fixed the hand in place. (3) Quantum random number generator (QRNG) sensor array-an array of 10 QRNG boards to measure entropic and negentropic effects were placed directly behind the head and torso of the participant and spatially throughout the room (results reported elsewhere). (4) Infrared camera- The infrared camera was placed two meters in front of the participant to optimally capture heat distribution changes in the participant’s face. Once participants were connected to all sensors, there was a 5-min stabilization period before the experiment began.

The experiment took approximately 70 min, however, only a portion of the experiment is reported here. Other experiment components, including affect-eliciting videos and a stress test will be reported elsewhere.

##### Data collection

Administration of the pre-survey, which included the PBBS, IOS, S-FoRC, NADA-state, and SAM was completed followed by a closed-eye baseline for 2 min. After the baseline, the guided loving kindness meditation was administered with participants’ eyes closed. Following the meditation, another baseline sensor measure with eyes closed was obtained for 2 min. A post-meditation survey was then administered with the same self-report questionnaires. The breathwork practice was then administered for 11 min. Immediately following the intervention, the questionnaires were administered again (PBBS, IOS, S-FoRC, the NADA-state, SAM). After the sensors were removed, qualitative data was collected from participants to learn more about the individuals experience with the meditation and breathwork interventions as well as well as their reactions to engaging in them.

### Statistical analysis

#### Self-report measures

Paired samples t-tests were performed to evaluate changes in self-report measures from pre to post. Statistical significance was set to *p* < 0.05. Questionnaire data were analyzed using SPSS version 22.0 (IBM).

#### Biofield measures

##### ECG, EDA, and EEG

Data processing was done using MATLAB R2021a (Mathworks, Natick, MA, United States). ECG measurements were processed to acquire HR. ECG was first high-pass filtered at 0.5 Hz, after which the QRS complexes of the ECG were detected using the Pan-Tompkins algorithm (Pan and Tompkins, 1985). The resulting semi-timeseries of consecutive inter-beat intervals was inverted to obtain HR and interpolated at 4 Hz.

Heart rate variability (HRV) was quantified using the root mean square of successive differences between normal heartbeats. This measure was computed using a moving window approach with a window size of 90 s and a step size of one second. EDA data was down-sampled to 16 Hz. The fast changing phasic (skin conductance response; SCR) component of the signal was extracted using Continuous Decomposition Analysis as implemented in the Ledalab toolbox for MATLAB (Benedek and Kaernbach, 2010). SCR more closely reflects sudomotor nerve bursts that stimulate the sweat glands (Benedek and Kaernbach, 2010), and better reflect psychological stress (Brouwer et al., 2017), compared to the raw signal or its slowly varying tonic component (skin conductance level; SCL). Therefore, we focus on SCR.

EEG was first down sampled at 256 Hz and high-pass filtered at 1 Hz. Data were then lowpass filtered at 47 Hz to remove line noise. Artifacts from the data were then removed using the clean_rawdata algorithm as implemented in EEGLAB for Matlab. Logistic infomax independent component analysis (ICA) was performed to identify the unique sources underlying the data in the 32 channels. The iclabel automatic ICA component labeler was used to identify artifactual independent components related to ocular or muscular activity, after which these components were removed from the data. The power spectral density in the Alpha band was computed separately for frontal (electrodes F3, F4, F7, F8) and occipital cortex regions (O1, Oz, O2). The power spectral density in the Alpha band was computed by averaging the power of the EEG signals across the frequency band from 9 to 12 Hz.

##### Infrared

Data processing was done using Python 3 including several common packages: data extraction was done using a pre-trained deep learning algorithm trained on infrared data (Kuzdeuov et al., 2022). The source of the infrared data was the infrared video recorded using a FLIR A655sc, which records temperatures with an accuracy of <30 mK. The time resolution of the camera was 25 Hz but was downsampled during data processing to 12.5 Hz. The resolution of the infrared video is 640×480 pixels. Data extraction was done by using a pre-trained deep learning algorithm to detect 54 landmarks on the face of the participant. Based on the locations of these points, five areas of interest (AOI) were determined.

Each AOI contained multiple pixel values (temperatures) at all times. The average and median of the temperature inside the AOI was analyzed initially. As average temperature was sensitive to facial movements and face tracking errors, all analyses were performed using the median temperature inside the AOI. The processed data was thus in the form of median temperature traces inside each AOI over time. The median temperature was tracked across the intervals of interest both as raw (median) temperatures and as normalized temperatures. To examine the effects, we normalized the temperature over the interval of interest by calculating the mean of the median temperature over the two seconds before the onset of the intervention and then subtracting this mean from the entire median temperature trace (Stuldreher et al., 2020). As a result, the normalized temperatures all start around 0 and show a (positive or negative) deviation from the initial median temperature. When inspecting the first results we found artifacts in the data due to improper tracking of the face when participants moved their heads, especially when looking downwards. To circumvent this a simple yet effective test was implemented to detect.

sudden and large movements of the detected landmarks from one frame to the next. These large movements are due to improper facial tracking. Participants for whom this happened often (>15% of the time during the interventions) were excluded from data analysis. Three participants were excluded from data analysis based on this threshold.

##### Ultraweak photon emission (UPE)

Photon emissions are recorded as the number of photons counted in 50 ms intervals. The counts are then averaged using a 5-min window with a moving step of one second, resulting in a smoothed trace over time, sampled at 1 Hz where the values represent the average photon count per 50 ms over the period t ± 2.5 min, where t denotes the time of the calculated value. During the moving average computation, all the extreme values, deviating over 15 times the standard deviation from the mean, were replaced by the mean. Then the mean and variance were recalculated. A representative trace for biophoton emission from both hands during meditation is provided in Supplementary Figure 1. For interventions that lasted 4 min or longer, averages were computed and statistically compared. Shorter interventions were deemed too short for analysis given the 5-min smoothing window. Analysis is done on a per-participant and per-intervention basis by subtracting the averaged photon count at the start of the intervention, for that participant, for the duration of the intervention. Therefore, the analysis examines the variation in photon emission relative to the onset of the intervention.

The above formulation largely eliminates the effects of dark noise, as this can be modelled by a negative binomial with parameters ${\hat{\mu }}_{\text{left}}\approx 9.2$ , ${\hat{\alpha }}_{\text{left}}\approx 0.47$ (Pearson χ^2^ = 1.31) and ${\hat{\mu }}_{\text{right}}\approx 5.7$ , ${\hat{\alpha }}_{\text{right}}\approx 0.31$ (Pearson χ^2^ = 1.17). Over the 5-min averaging window (6,000 bins), these would result in standard deviations of $SD_{\text{left}}\approx 0.09$ and $SD_{\text{right}}\approx 0.05$ . On long time scales (>5 min) these would increase by a factor $\sqrt{2}$ to account for the independent noise in the subtracted baseline.

##### Effects of mind–body practices on biofield measures

To examine the effects of meditation and breathwork practice on biofield measures, we baselined all signals by subtracting the average value of the two seconds before the onset of the intervention. This effectively shifts the traces to start at zero and reduces effects of slow drifts and (assumingly, irrelevant) differences between participants in the measures’ absolute values. Next, traces were averaged across participants. For meditation and the breathwork exercises, we compared average values during the interventions against the preceding resting baseline, using paired t-tests.

##### Individual changes in biofield measures

Boxplots for each biofield measure were generated to assess individual differences in participants’ responses to the meditation and breathwork.

##### Correlational analysis of biofield measures

An exploratory analysis into potential within-subject correlations between biofields was conducted. The Pearson correlation coefficient was calculated for each pair of biofields. Due to the sampling rate and duration of the measurements, on initial inspection, many of the measured signals showed strong significance but low correlation. To filter the signals which correlated strongly, the mean correlation coefficient was calculated using a Fisher Z-transform to convert the Pearson correlation coefficients to a normally distributed variable (Pinelis, 2019). The Fisher Z-transform was applied to ensure the mean correlation coefficient reflected the strength of the correlations accurately, accounting for the variability and mixed direction of the effects.

## Results

### Feasibility

#### Recruitment and retention

**Figure 1:**
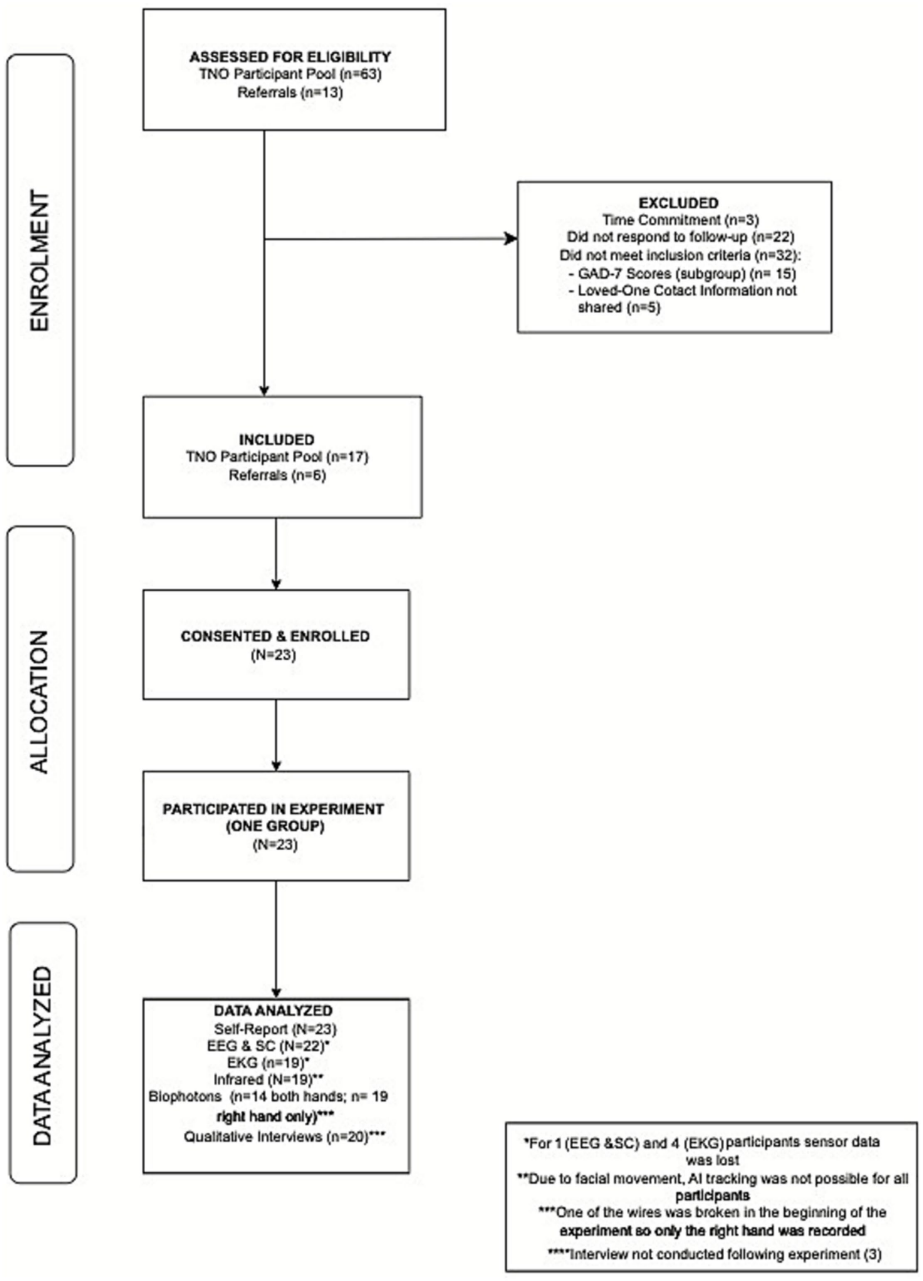
CONSORT diagram.

Recruitment was achieved within the estimated timeframe of 1.5 months (May 1–June 15, 2022) and all 23 participants who agreed to participate completed the study (100% retention). Further details are displayed in the CONSORT diagram in Figure 1.

#### Data completeness

There was no missing self-report data. As mentioned previously, when inspecting the first results we found several artifacts in the IR data due to improper tracking of the face when participants moved their head, especially when looking downwards. To circumvent this a simple yet effective test was implemented to detect sudden and large movements of the AOI’s from one frame to the next. These large movements are due to improper facial tracking. Participants for whom this happened often (>15% of the time during the interventions) were excluded from data analysis. As a result, three participants were excluded from data analysis based on this threshold (13% of participants).

For the UPE measurements only the right hand was recorded in 5 participants due to a broken wire and 4 participants’ UPE data was lost due to experimental lead error (17% of participants).

#### Acceptability

Despite the extended duration of the experiment and the requirement for participants to remain still with sensors attached and both hands fixed at all times, no participants reported issues with the duration of their involvement or any significant discomfort. However, six participants expressed that they did not enjoy the meditation intervention, primarily citing difficulties with concentration and challenges in adhering to instructions to remain in the present moment or clear their minds of extraneous thoughts. Additionally, four participants felt that the meditation intervention was excessively long, resulting in a loss of focus towards the end. In contrast, the breathwork intervention received more favorable feedback. Although five participants initially felt overwhelmed by the task of breath-holding, they were eventually able to comply with the instructions. Overall, the breathwork intervention was generally well-accepted, whereas some participants did not enjoy or fully adhere to the meditation intervention.

### Self-report outcomes

#### Meditation

Following the loving kindness meditation, participants reported significantly lower.

arousal (SAMA) (*p* = <0.001), increased sense of dominance (SAMD) (*p* = 0.012), but no changes in valence (SAMV) (*p* = 0.118) compared to the pre-meditation baseline. Participants’ perception of how far their sense of self (the field around their body) extends into the word (S-FoRC) was significantly increased (*p* = 0.011), and their state non-duality (stronger sense of unity with the world) (NADA-S) (*p* = < 0.001), but no change in boundary dissolution (PBD) (*p* = 0.169), or their feeling of connectedness to others (IOS) (*p* = 0.123) (see Table 2).

**Table 2:** Means and standard deviations and statistics of self-report data at pre and post meditation and breathwork.

Measure	Meditation				Breathwork			
	Pre	Post	d	*p*	Pre*	Post	d	*p*
SAMA—arousal	3.22 (1.26)	2.00 (1.17)	0.825	**<0.001**	2.00 (1.17)	2.87 (1.71)	0.409	**0.011**
SAMD—dominance	6.13 (1.74)	6.61 (1.72)	0.505	**0.012**	6.61 (1.72)	6.83 (1.67)	0.268	0.248
SAMV—valence	6.83 (1.19)	7.17 (1.13)	0.254	0.118	7.17 (1.13)	7.17 (1.34)	0.074	0.500
S-FoRC—extension of self	3.39 (1.78)	4.30 (1.64)	0.513	**0.011**	4.30 (1.64)	3.39 (1.47)	0.619	**0.004**
IOS—connection with others	4.39 (1.53)	4.70 (1.40)	0.249	0.123	4.70 (1.40)	4.17 (1.53)	0.388	**0.038**
PBD—Perceived boundaries	3.91 (1.83)	4.22 (1.51)	0.204	0.169	4.22 (1.51)	4.13 (1.77)	0.080	0.352
NADA—state nonduality	12.00 (7.51)	16.74 (6.85)	0.969	**<0.001**	16.74 (6.85)	15.04 (7.52)	0.884	**0.039**

Significant results are in bold. *Pre-breathwork is the same as post-meditation.

#### Breathwork exercise

The breathwork exercise resulted in a significant increase in arousal (SAMA) (*p* = 0.011) but had no effect on valence (SAMV) (*p* = 0.248) or dominance (SAMD) (*p* = 0.500). There was a decrease in the participant’s perception of how far their self of self extends into the world (S-FoRC) (*p* = 0.004), a decrease in their feeling of connectedness to others (IOS) (*p* = 0.038), and a decrease in state non-duality (NADA-S) (*p* = 0.039), but no change in perceived boundaries with the world (PBD) (*p* = 0.352) when compared to the post-meditation, pre-breathwork exercise baseline (see Table 2).

A summary of the changes in self-report measures (e.g., increase, decrease, or no change) following the meditation and breathwork exercise is displayed in Table 3.

**Table 3:** Summary of self-report measures either significantly increasing (↑), decreasing (↓), or not changing (--) following each practice.

Self-report measure	Meditation	Breathwork
SAMA—arousal	↓	↑
SAMD—dominance/Control	↑	--
SAMV—valence	--	--
S-FoRC—extension of self	↑	↓
IOS—connection with others	--	↓
PBD—perceived boundaries	--	--
NADA—state nonduality	↑	↓

### Biofield measures

#### Meditation

**Figure 2:**
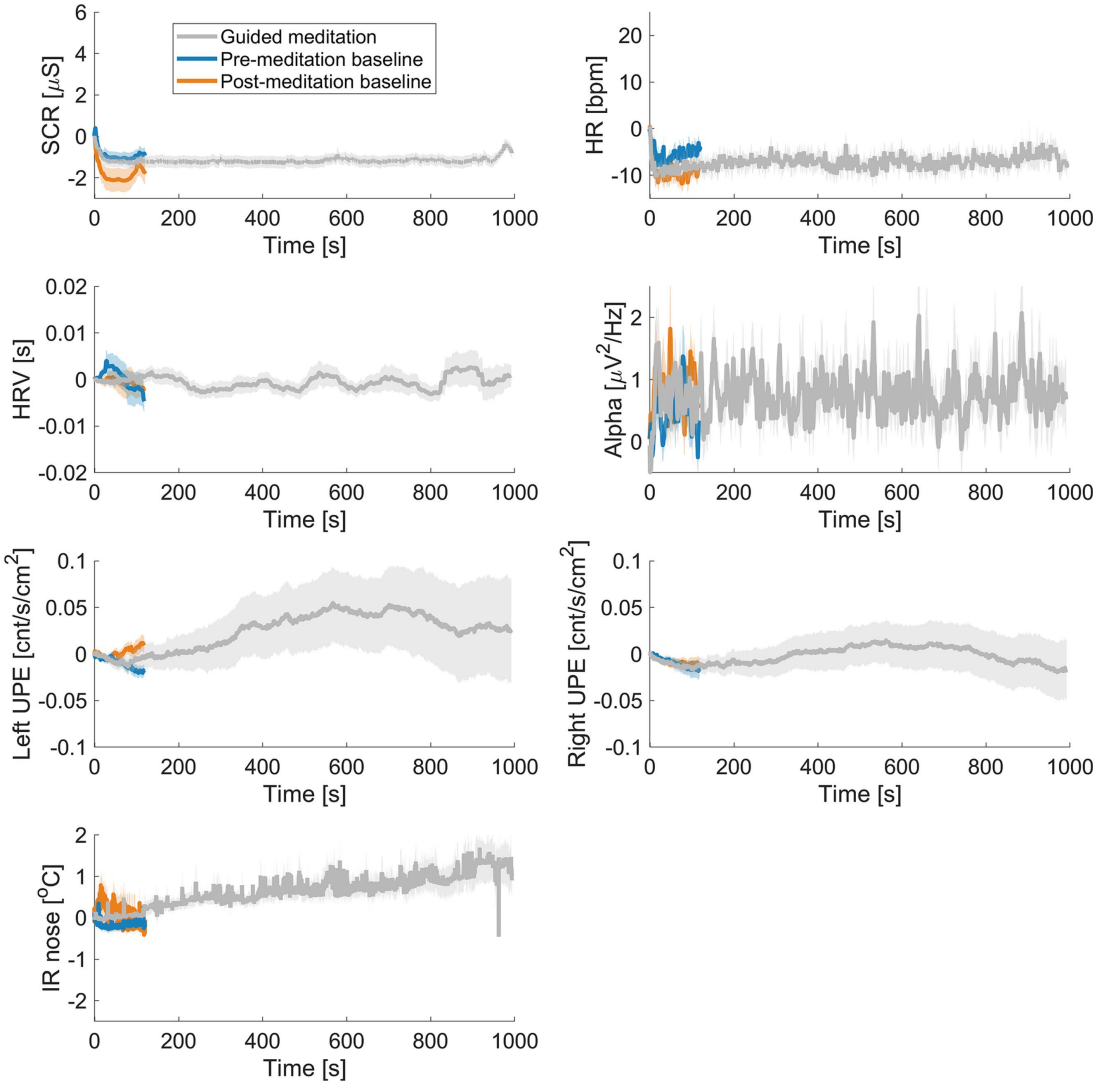
Time course averaged delta across all participants during the audio guided loving kindness meditation for skin conductance (SCR), heart rate (HR), heart rate variability (HRV), EEG alpha, ultraweak photon emission (UPE), and infrared (IR) of the nose.

Figure 2 displays the biofield-related measures over time during the audio guided loving kindness meditation, averaged across participants. For SCR, HR and HRV, no differences between baseline and meditation were observed. There was also no effect of meditation for alpha in the overall data. UPE intensity from the left hand increased to a maximum after about 10 min, and then decreased again. Deviations were only slightly larger than expected dark noise fluctuations, accounting for the number of participants and the noise in the subtracted baseline ( $SD_{\text{dark}-\text{left}-N=15}\approx 0.033$ and $SD_{\text{dark}-\text{right}-N=19}\approx 0.016$ ). Paired-sample t-tests comparing the mean UPE intensity in the first 10 s of the meditation to the 10 s in the middle of the intervention did not reveal a significant difference (t (13) = 1.13, *p* = 0.277). Total UPE intensity was similar to previous research (Zhao et al., 2016) using the same type of PMTs: 52 counts per second on average. Correcting for the PMT quantum efficiency of 30% at peak, this shows approximately 173 photons arrive at the sensor per second. IR showed a clear increasing trend (*p* = 0.003). When comparing the values averaged across the whole meditation interval to baseline, values were significantly higher than baseline for IR (see Table 4).

**Table 4:** Results of the paired t-tests, comparing measures averaged across the condition intervals to the appropriate baseline intervals.

Measure	Baseline	Meditation	Meditation vs. Baseline	Baseline	Breathwork	Breathwork vs. Baseline*
SCR	−0.70 (0.93)	−1.10 (1.21)	t(21) = −1.68, *p* = 0.109	−1.66 (2.09)	−1.07 (1.45)	t(21) = 1.70, *p* = 0.104
HR	−5.80 (5.39)	−6.84 (7.40)	t(18) = −0.59, *p* = 0.565	−8.77 (6.72)	−1.95 (4.96)	**t(18) = 3.22, p = 0.005**
HRV	0.00 (0.01)	−0.00 (0.07)	t(18) = 0.34, *p* = 0.741	−0.00 (0.01)	−0.01 (0.02)	t(18) = −1.91, *p* = 0.073
EEG alpha	0.46 (0.97)	0.72 (1.42)	t(21) = 0.847, *p* = 0.394	0.74 (0.93)	0.53 (0.76)	t(21) = −1.02, *p* = 0.318
UPE-L	−0.01 (0.02)	0.03 (0.12)	t(13) = 1.15, *p* = 0.270	0.00 (0.02)	−0.05 (0.09)	t(13) = −2.09, *p* = 0.057
UPE-R	−0.01 (0.02)	−0.00 (0.08)	t(18) = 0.42, *p* = 0.679	−0.01 (0.02)	−0.02 (0.07)	t(18) = −0.70, *p* = 0.492
IR	−0.04 (0.21)	0.73 (0.84)	**t(20) = 3.85, *p* = 0.001**	0.19 (0.91)	−1.41 (1.06)	**t(20) = −5.87, *p* < 0.001**

Significant results are in bold. *Baseline is post-meditation assessment.

#### Breathwork exercise

**Figure 3:**
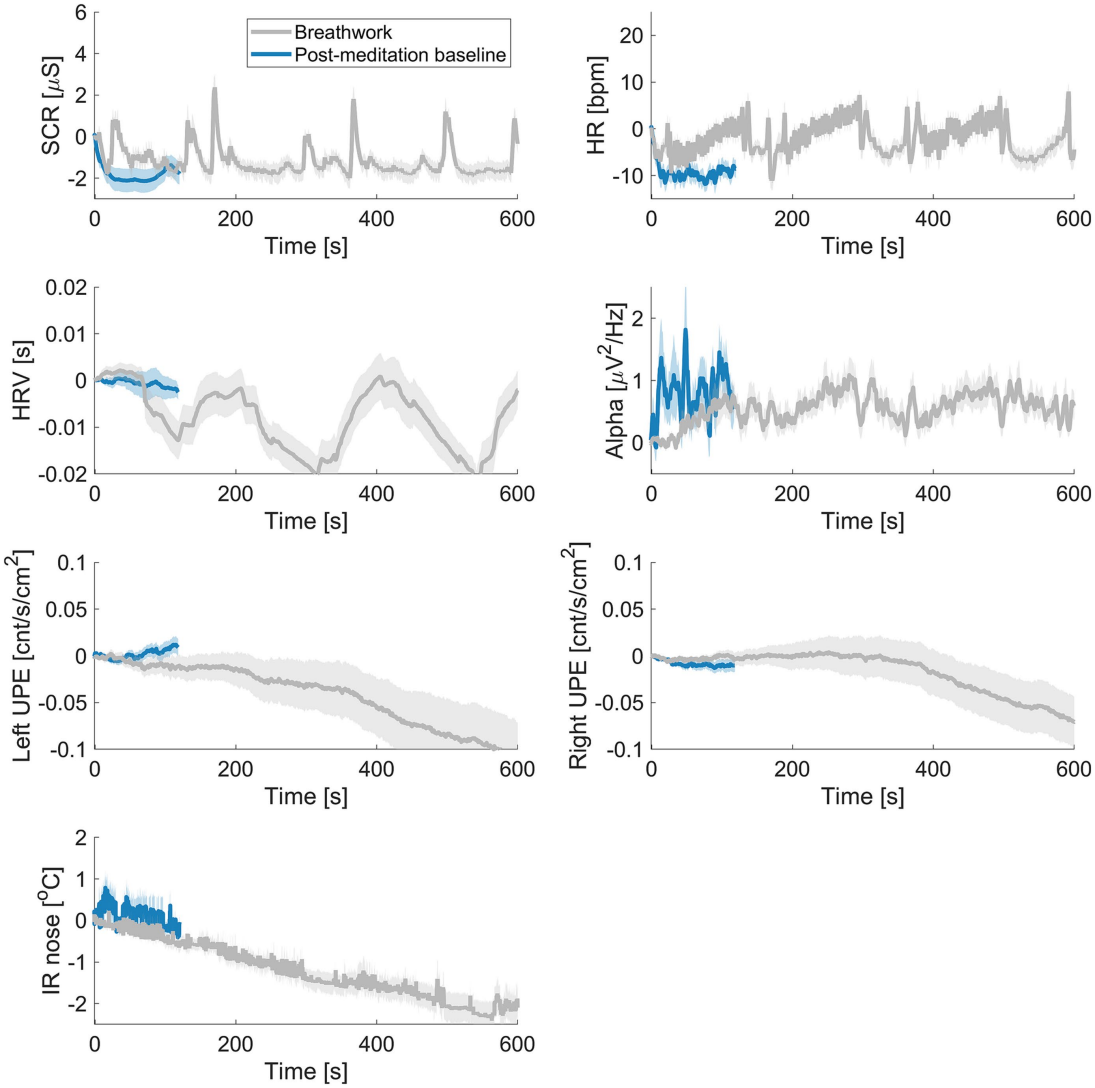
Time course averaged delta across all participants during the breathing exercise for skin conductance (SCR), heart rate (HR), heart rate variability (HRV), EEG alpha, ultraweak photon emission (UPE), and infrared (IR) of the nose.

Figure 3 displays the biofield-related measures over time during the breathwork exercise, averaged across participants. The on-body biofield measures, EDA, HR, HRV and alpha showed a modulation over time in line with the instructed breathing pattern (three intervals of vigorously breathing followed by holding the breath). HR was significantly higher than baseline (*p = 0.005); but the other measures did not show a significant overall effect. UPE showed a decreasing trend with the overall effect close to significance (*p* = 0.057) for the left UPE. Deviations were larger than expected dark noise fluctuations. IR strongly decreased, resulting in a significant difference (*p* < 0.001) between breathwork interval and baseline (see Table 4).

#### Individual responses to meditation and breathwork interventions

**Figure 4:**
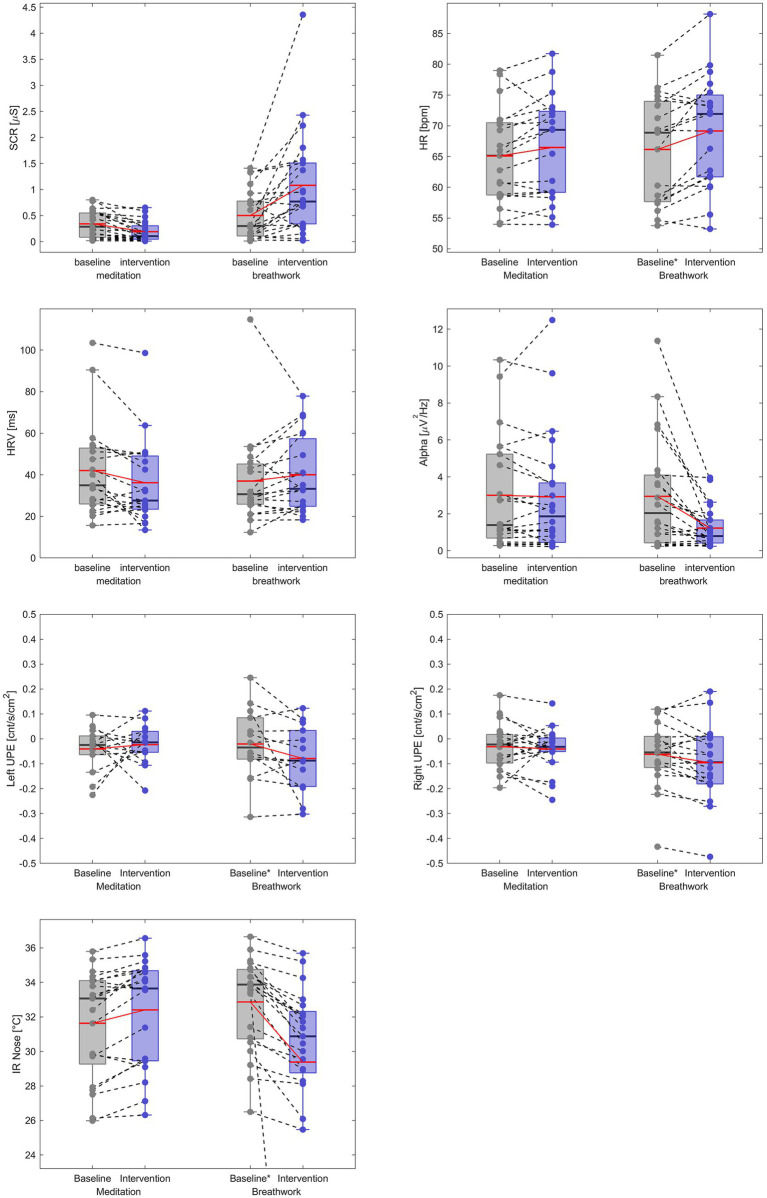
Boxplots of individual biofield responses pre- and post meditation and breathwork interventions.

Inspection of the individual responses in the box plots (Figure 4) revealed that some participants’ biofield measures changed throughout the experiment as expected for the meditation and breathwork, whereas others did not. In some cases, changes were observed in the opposite direction as expected for the interventions. Table 5 summarizes the number of participants that showed either an increase (−↑) or a decrease (↓) on each biofield measure for the meditation and breathwork.

**Table 5:** Number of participants that showed an increase (↑) or decrease (↓) on each biofield measure for the meditation and breathwork exercises.

Biofield measure	Meditation		Breathwork	
	↑	↓	↑	↓
SCR (*n* = 22)	10	12*	14*	8
HR (*n* = 19)	9	10*	14*	5
HRV (*n* = 19)	11*	8	6	13
EEG alpha (*n* = 22)	10	12	12	10
UPE Left (*n* = 14)**	9	5*	4	10
UPE Right (*n* = 19)**	11	8*	9	10
IR Nose (*n* = 21)	18*	3	0	21

*Indicates the anticipated direction (increasing or decreasing) based on previous research. **Previous research in advanced meditators has found decreases in UPE, but no studies have been performed with a sample with a majority of novice meditators.

#### Correlation analysis

##### Meditation

**Figure 5:**
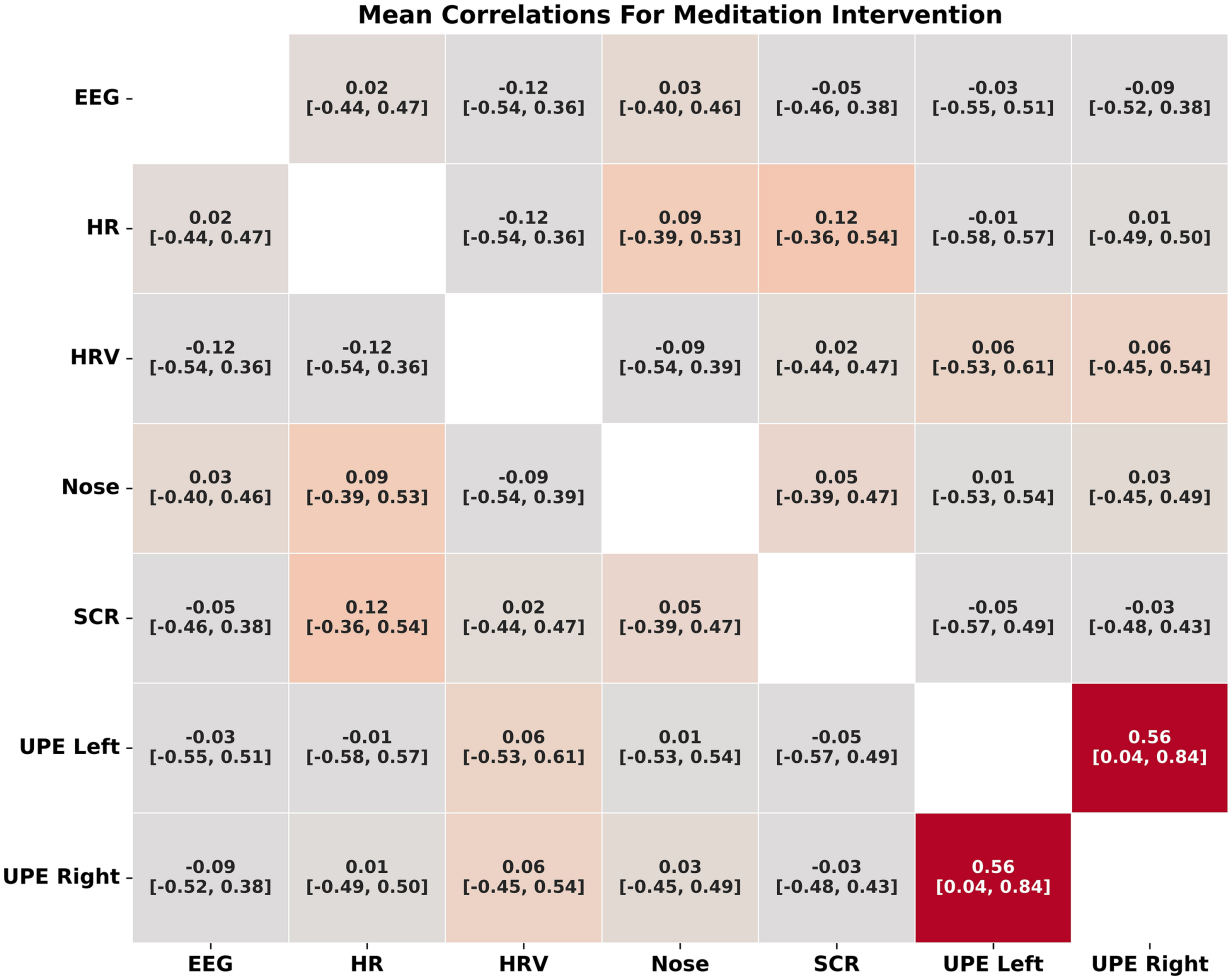
Correlations between biofield measures for the meditation intervention.

The correlational analysis for the meditation intervention revealed strong correlations between biofield measures of UPE from the left and right hands (r = 0.56, *p* = <0.001, 95% CI [0.04, 0.84]) (Figure 5 for correlations and Supplementary Figure 2 for *p* values). Note that the correlation in UPE cannot be explained by dark noise alone. No other measures had strong or moderate correlations. All the correlations were weak (r < 0.3) but were significant (*p* < 0.02). Given that more than a third of the participants reported that they did not engage in the meditation, we also filtered these nine from the analysis to see if this impacted the correlations. Filtering these nine participants strengthened the already strongly correlated UPE left and right hand (r = 0.66, *p* < 0.001, 95% CI [−0.01, 0.92]) and strengthened the correlation between UPE left and IR nose temperature to an almost moderate correlation (r = 0.28, *p* < 0.001, 95% CI [−0.07, 0.80]) (Supplementary Figure 3). Although all other correlations increased after filtering the participants who did not engage, this did not result in any other measures correlating moderately or strongly.

When all the measures were transformed (using the Fisher z transform) and the absolute value were assessed for their correlations there was a strong correlation between UPE from the left and right hands (r = 0.58) and moderate correlations between UPE left hand and IR nose temperature (r = 0.45) and UPE right hand and IR nose temperature (r = 0.45), and UPE left hand and HRV (r = 0.34) (Supplementary Figure 4).

##### Breathwork

**Figure 6:**
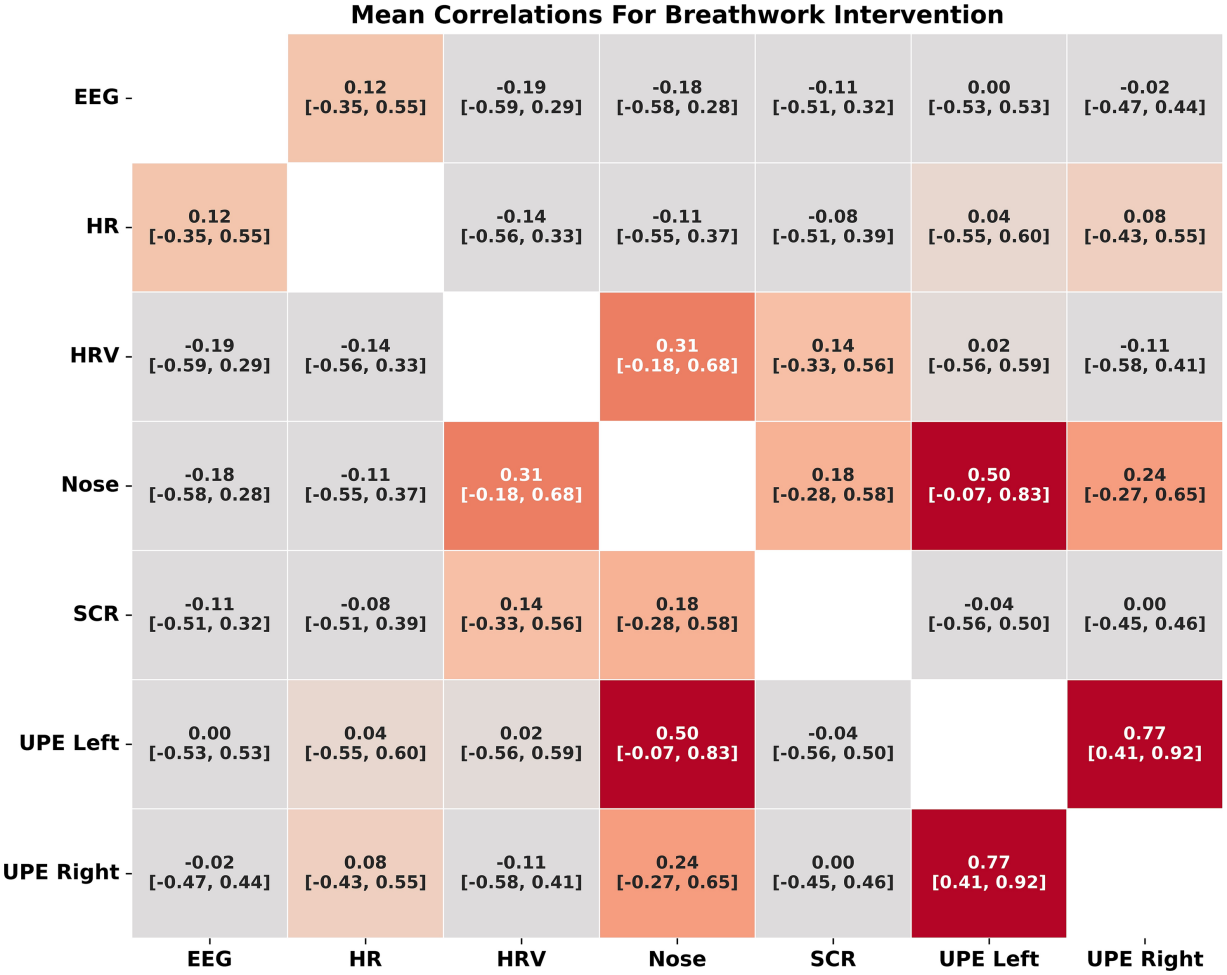
Correlations between biofield measures for the breathwork intervention.

Similarly, the breathwork intervention demonstrated strong correlations between left and right hand UPE (r = 0.77, *p* < 0.001, 95% CI [0.41, 0.92]) and between IR nose temperature and left hand UPE (r = 0.50, *p* < 0.001, 95% CI [−0.07, 0.83]). Moderate correlations were found between IR nose temperature and HRV (r = 0.43 *p* = <0.001, 95% CI [−0.18, 0.68]). All other measures showed weak correlations (r < 0.3) but were significant (*p* < 0.001) (Figure 6; Supplementary Figure 5).

When all the measures were transformed (using the fisher z transform) and the absolute value were assessed for their correlations there remained a strong correlation between UPE from the left and right hands (r = 0.78), UPE left hand and IR nose temperature (r = 0.75), UPE right hand and IR nose temperature (r = 0.68). Moderate correlations were also established between IR nose temperature and HRV (r = 0.43), UPE right hand and HRV (r = 0.38) and UPE left hand and HRV (r = 0.37) (Supplementary Figure 6).

##### Qualitative interviews

Qualitative interviews (*n* = 20) indicated that some participants reported a lack of familiarity with meditation (*n* = 9) and/or breathwork (*n* = 13), and a subset reported being unable to engage with either the meditation (*n* = 9) or breathwork interventions (*n* = 4). Conversely, some participants had experience with meditation (*n* = 11), with a subset of these participants regularly engaging in a meditative practice (*n* = 3). Some participants had previously practiced high ventilation breathwork (*n* = 7), although none practiced it regularly.

## Discussion

To our knowledge, this is the first study to simultaneously measure on-body and off-body biofield measures during a meditation and breathwork practice, and to measure IR and/or UPE during a breathwork exercise. This exploratory study demonstrated sufficient feasibility to continue the research with a larger sample size and appropriate controls. Of the 23 participants recruited all of them completed the study in its entirety (100% retention). It was feasible to record all measures simultaneously during the different interventions, and participants tolerated the study well.

### Data completeness

In terms of data completeness, two issues were encountered: the complicated nature of the experimental set-up and artifacts in the IR data, primarily due to improper tracking of the face when participants moved their heads, especially when looking downwards. Due to the large volume of data being collected, issues such as broken wires were not identified and repaired quickly enough, so some data was lost. As for the IR data artifacts, much of this was due to improper tracking of the face when participants moved their heads, especially when looking downwards. Participants for whom this happened often (>15% of the time during the interventions) were excluded from data analysis, which were three participants.

### Meditation and breathwork

The meditation and breathwork practices resulted in nearly contrary patterns with respect to both self-report and biofield measures. Self-reports indicate that the loving kindness meditation induced low arousal, a greater sense of dominance or control, and feelings of unity with the world and others. Loving kindness meditation has been shown to increase feelings of connectedness in other studies (Hutcherson et al., 2008; Adventure-Heart and Proeve, 2017; Don et al., 2022). In contrast, the breathwork exercise was associated with high arousal and a focus on the self. Although there have not been any studies investigating changes in states of consciousness (such as nonduality and perceptions of boundaries) for the WHM, there has been research into other high ventilation breathwork practices that contain similar features (Fincham et al., 2023). Most high ventilation breathwork practices have their origins in and were a component of ancient rituals or practices for spiritual and healing purposes across many different cultures some dating back 10,000 years and designed to induce altered states of consciousness (Brown and Gerbarg, 2012; Deepak, 2002; Grof and Grof, 2023; Raghuraj et al., 1998).

Research to date has found that other high-ventilation practices (particularly holotropic breathwork) can lead to altered states of consciousness (Fincham et al., 2023; Grof and Grof, 2023). However, the appearance of these states is usually a consequence of the breathwork practice and is not experienced during breathwork exercise itself. In future studies looking at the WHM, it would be important to better understand when the changes in states of consciousness occur as to time the collection of the self-report measures to adequately understand how the exercise may be affecting the participant’s conscious state through the practice (Fincham et al., 2023). Also, further study is needed to understand the effects of the sequence of interventions, and the timing between them.

During the breathwork exercise, EDA, HR, HRV and EEG alpha showed a modulation over time in line with the instructed breathing pattern, reflecting the three cycles of vigorous breathing and holding the breath. Overall, averaged across the exercise, EDA increased and HRV decreased but only with marginal significance (*p* = 0.061 and *p* = 0.071 respectively). These physiological findings are in line with the observed increase in self-reported arousal. However, note that we cannot interpret these findings as a consequence of alterations in mental state since breathing by itself has profound effects on physiology.

No changes in EEG alpha due to meditation were observed, particularly in prefrontal alpha wave asymmetry, as reported in other studies of loving kindness meditation and associated with relaxed attention and reduced arousal (Barnhofer et al., 2010; Yordanova et al., 2020). This may be due to lack of experience with or participation in the mediation practice as reported by participants in the qualitative interviews (*n* = 9).

The most prominent change for the meditation and breathwork was observed for IR. The loving kindness meditation was associated with significantly higher IR of the nose compared to baseline. A previous study of loving kindness meditation also found an increase in nose temperature following 10 min of meditation, but it did not significantly differ from nose temperature from 10 min of resting (Wang et al., 2023). In contrast to the meditation, IR of the nose was significantly lower during the breathwork exercise compared to the post-meditation, pre-breathwork baseline. This could be due to increased airflow cooling the nose during inspiration (Pereira et al., 2015) or from an emotional/mental cause (An et al., 2022; Clay-Warner and Robinson, 2015), but more research is needed to confirm or refute these ideas.

Meditation seems to influence the complex interactions of oxidative and anti-oxidative reactions which regulate photon emission (Van Wijk et al., 2005) reporting that experienced meditators (at least 15 years of meditating) showed decreased UPE of the hand and forehead during meditation. However, this study had only 5 subjects, a much smaller sample than the current study. Similarly, a study with transcendental meditation reported decreased UPE in the hand compared to controls (Van Wijk et al., 2008). In this light, it is unclear why we did not observe significant overall decreases in UPE during meditation in the current study. It is noteworthy that the meditation and breathwork were both single sessions delivered to inexperienced participants. Meditation and breathwork are practices that are meant to be performed regularly over time. Therefore, it could take a longer time to observe physiological benefits or changes with these two practices. Future work should evaluate changes in UPE over time with continued practice.

While UPE was not significantly different overall from baseline for either practice, the breathing practice resulted in a decrease in UPE of the left hand that was close to significance (*p* = 0.057). Wim Hof’s stimulating breathing exercise involves both hyperventilation (possibly inducing oxidative stress) and breath retention (possibly inducing hypoxia). Physiologically, the oxygen level in blood is in saturation (close to 100%). Therefore, the influence of hyperventilation is limited. Hypoxia may have two impacts on mitochondria: reducing the oxidative stress and increasing the efficiency of mitochondria (similar to high-altitude adaptation of human). Both effects may cause a reduction in UPE. No previous research on UPE and this type of breathwork has been conducted to allow for comparison with the literature. Therefore, much more research is needed in this area to elucidate a possible association between WHM breathing and UPE.

### Individual responses

Averaged responses to the interventions, particularly the meditation, did not align with previous research findings. Some participants’ biofield measures changed as expected throughout the experiment, others did not change, and others changed in the opposite direction as expected. Reasons for these mixed results are unknown but could be due to variability in participants’ previous experience and/or engagement in the practices. However, some participants that did not have prior experience with meditation were still able to engage in the practices and breathwork practices, which was reflected in their outcomes.

Because our recruitment strategy aimed to identify healthy participants regardless of their experience with meditation and/or breathwork, this heterogeneity likely contributed to the mixed responses. For example, in support of this contention, previous studies have reported that those with experience in meditation differ in UPE from those without experience in meditation (Van Wijk et al., 2006, 2008). Regardless, preliminary evidence from the current study suggests that when experienced participants properly engaged in the practices, changes in their biofield measures were consistent with previous studies. These results demonstrate the importance of tracking engagement with the intervention, and future work will aim to recruit participants with experience in meditation to insure consistency. We chose practices that were relatively well-known and shown to be effective at altering mental state. Future work could investigate other practices using the same outcome measures.

### Correlational analysis

The exploratory analysis of within-subject correlations between biofields revealed several insights and highlighted key considerations for future research. Calculating the Pearson correlation coefficient for each pair of biofields showed a substantial portion of significant correlations, though many had low values, potentially misleading due to the signal length and nature. Despite these limitations, the analysis indicated strong underlying correlations among biofield signals, suggesting common physiological or biofield mechanisms. This interconnectedness is promising for understanding biofield dynamics.

A primary challenge was analyzing minimally processed signals. For example, heart rate (HR) and heart rate variability (HRV) are inherently correlated, representing different aspects of the same physiological process. However, without processing to extract specific features, the true nature of their relationships may have been conflated.

Using the Fisher Z-transform to calculate the mean correlation coefficient normalized the Pearson correlations, highlighting strong within-subject correlations with mixed directions of effect. While all signals were significant, the correlation values suggest that mere statistical significance might not accurately reflect the true nature of the correlations due to the long measurement duration.

These findings underscore the need for more sophisticated methods in future research. Developing precise signal processing techniques that account for the unique processing and acquisition times of each signal type will be crucial. This will help isolate the underlying factors driving correlations and provide a more accurate representation of biofield relationships.

This analysis demonstrated a few strong underlying correlations, indicating interconnectedness within some of the measurements. Future work should refine these methods to enhance the reliability and interpretability of observed correlations, improving our understanding of biofield dynamics and their relationship to mental state.

### Limitations

As this was a highly exploratory study, there were several limitations. First, all participants received meditation before the breathing exercise which was always performed last because vigorous breathing was expected to strongly affect physiology and necessitate a long subsequent resting period to return to baseline values. Because the order of meditation and the breathing exercise was not cross balanced, we cannot directly compare the magnitude effects of meditation on self-report and biofield measures to that of the breathing exercise, due to possible order effects. Second, this study was part of a larger study and as such, the participants received additional interventions before the meditation, including emotion elicitation and stress inductions. Therefore, we are not able to ascertain what effects may have been carried over from the previous exercises, despite there being short resting periods in between. Future work will avoid having participants perform multiple practices during the same experiment.

Third, this exploratory study did not have a control group, limiting our ability to draw conclusions from the findings. It is not possible to determine, for example, whether changes observed were due to specific effects of the practices themselves or nonspecific effects such as time, expectations, or the Hawthorne effect. Fourth, as a feasibility study there was no power calculation performed and as such, the sample size was likely insufficient to detect meaningful differences. Future work with a fully powered, larger sample size will address this limitation.

Lastly, the correlational analysis was performed on limitedly processed signals, looking at different aspects of human physiology, and not processed in a way that would suggest any correlations from a mechanism perspective. Therefore, except for the UPE in the left and right hand, there was no prior reason to expect correlations, which may affect the reliability of the results. This exploratory analysis was designed to provide an initial look into potential correlations to explore in future research.

## Conclusion

The current study demonstrated that it was feasible to simultaneously record with multiple on and off body sensors to measure electromagnetic fields and currents while subjects were engaged in mind and body practices. Beyond study feasibility, the self-report and biofield measure results are highly preliminary due to participant heterogeneity and low sample size. Therefore, they should be interpreted as such and re-examined in future research. The meditation practice led to decreased arousal, increased unity with others and the world, and nonduality, whereas the breathing exercise led to increased arousal and focus on the self. Meditation led to significant increased IR of the nose, whereas the breathing practice led to a decrease in IR of the nose. There were also nearly significant increases in HR, HRV, and UPE for the breathwork practice. There were individual differences in participants’ biofield measure changes, where some change as expected throughout the experiment, others did not. Reasons for these mixed results might be due to variability in participants’ previous experience and/or engagement in the practices.

These preliminary findings, while highly exploratory, provide support to continue to a randomized controlled trial with a more homogenous population experienced in meditation and/or breathwork, and with a particular focus on measuring IR and UPE. Further, the correlational analysis, although highly preliminary, indicated strong underlying intra-subject correlations among different biofield signals, suggesting common physiological or biofield mechanisms. Moreover, effects of varying the sequence, intensity, and duration of the practices could also be explored in subsequent studies. This interrelatedness is promising for understanding the potential systemic nature of biofield dynamics and will be investigated in future research.

## Data Availability

The original contributions presented in the study are included in the article/Supplementary material, further inquiries can be directed to the corresponding author.

## Supplementary material

SUPPLEMENTARY FIGURE 1 A representative trace of biophoton emission from both hands during meditation.

SUPPLEMENTARY FIGURE 2 *P* values from the correlational analyses for biofield measures from the meditation intervention.

SUPPLEMENTARY FIGURE 3 Correlations (r) between biofield measures from the meditation intervention after filtering out the nine participants that did not engage in the meditation.

SUPPLEMENTARY FIGURE 4 Correlations (r) between absolute values of biofield measures from the meditation intervention after the Fisher z transformation.

SUPPLEMENTARY FIGURE 5 *P* values from the correlational analyses for biofield measures from the breathwork intervention.

SUPPLEMENTARY FIGURE 6 Correlations (r) between absolute values of biofield measures from the breathwork intervention after the Fisher z transformation.

## Appendix

### Inclusion/exclusion criteria of all participants (*n* = 23)

Inclusion criteria:

- Comfortable communicating and completing questionnaires in English.
- Capable of seeing videos and reading text on a monitor without the use of glasses or can use contacts.

Exclusion criteria:

- Diagnosed with physical or mental health conditions.
- Recovering from an injury.
- Engages in regular recreational drug use.
- BMI > 25.
- The participant has a metal plate or bar implant in their body.
- Unable to see without the use of glasses and cannot use contacts.
- Regularly takes prescription medication (excluding the contraceptive pill).
- Has an active (i.e., currently presenting symptoms) skin condition or other skin problem (e.g., eczema, psoriasis).
- Regularly smokes cigarettes (including e-cigarettes).
- Consumes more than 28 units of alcohol a week (more than three bottles of wine).
- Pregnant, planning to become pregnant, or have had a child within the last 6 months, or are currently breast feeding a child.
