# Supplementary figures and images for: Changes in biofield measures and experienced states during meditation and breathwork practices: an uncontrolled feasibility study
Source: Front Psychol. 2026 Feb 25;17:1623301. doi: 10.3389/fpsyg.2026.1623301 (PMC12978019; doi:10.3389/fpsyg.2026.1623301)

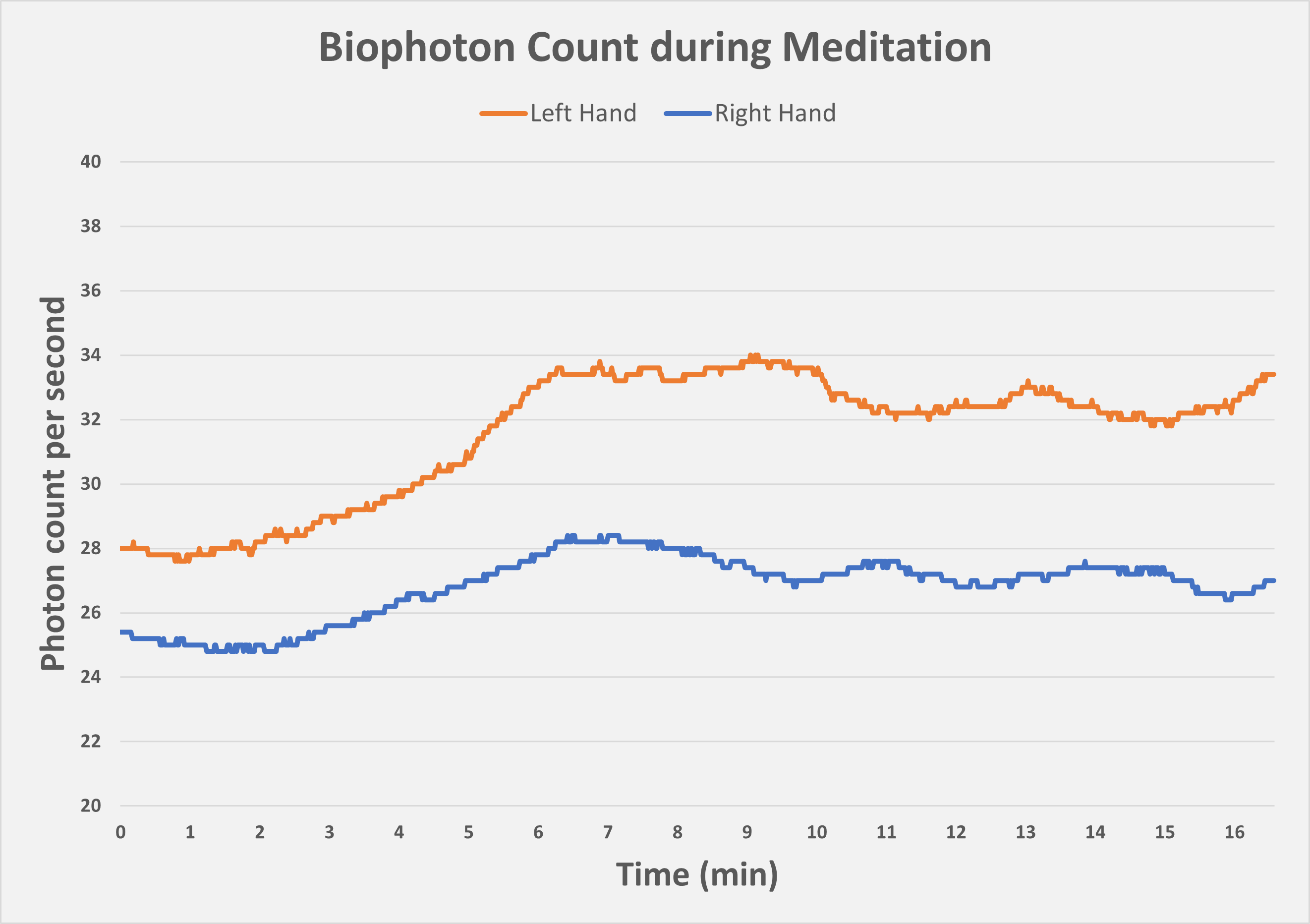

Supplement: SUPPLEMENTARY FIGURE 1 — A representative trace of biophoton emission from both hands during meditation. [file Image_1.png]

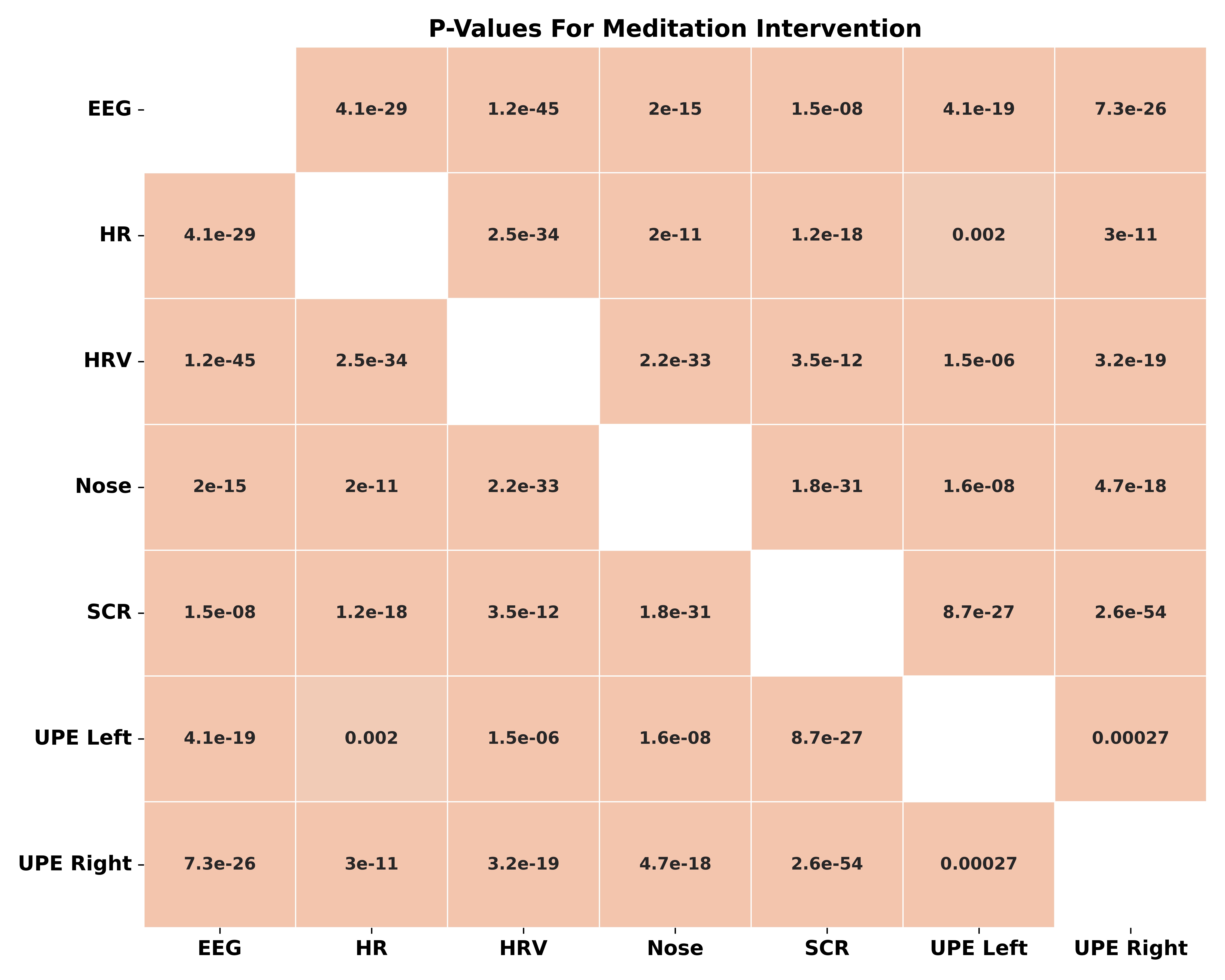

Supplement: SUPPLEMENTARY FIGURE 2 — P values from the correlational analyses for biofield measures from the meditation intervention. [file Image_2.jpeg]

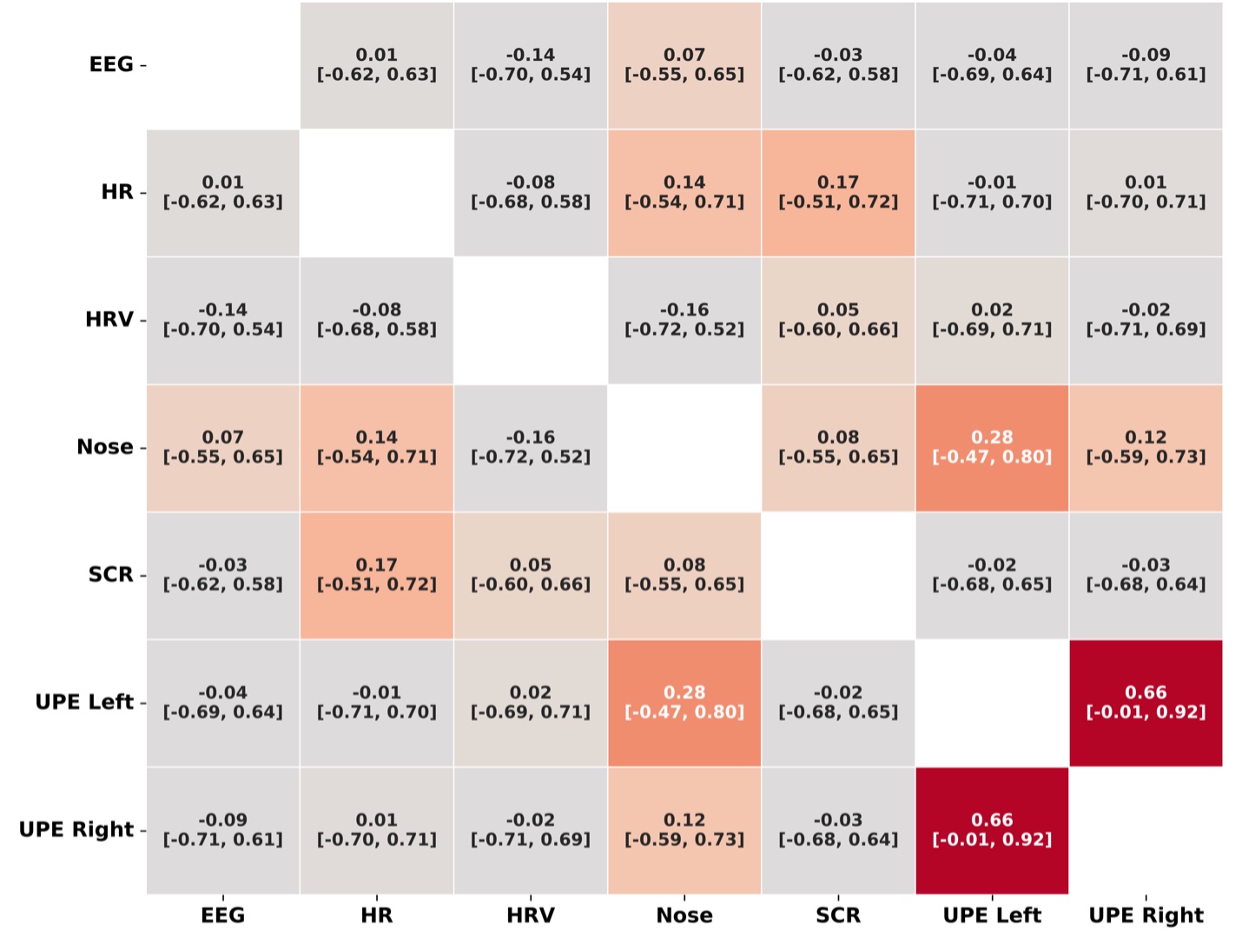

Supplement: SUPPLEMENTARY FIGURE 3 — Correlations (r) between biofield measures from the meditation intervention after filtering out the nine participants that did not engage in the meditation. [file Image_3.jpeg]

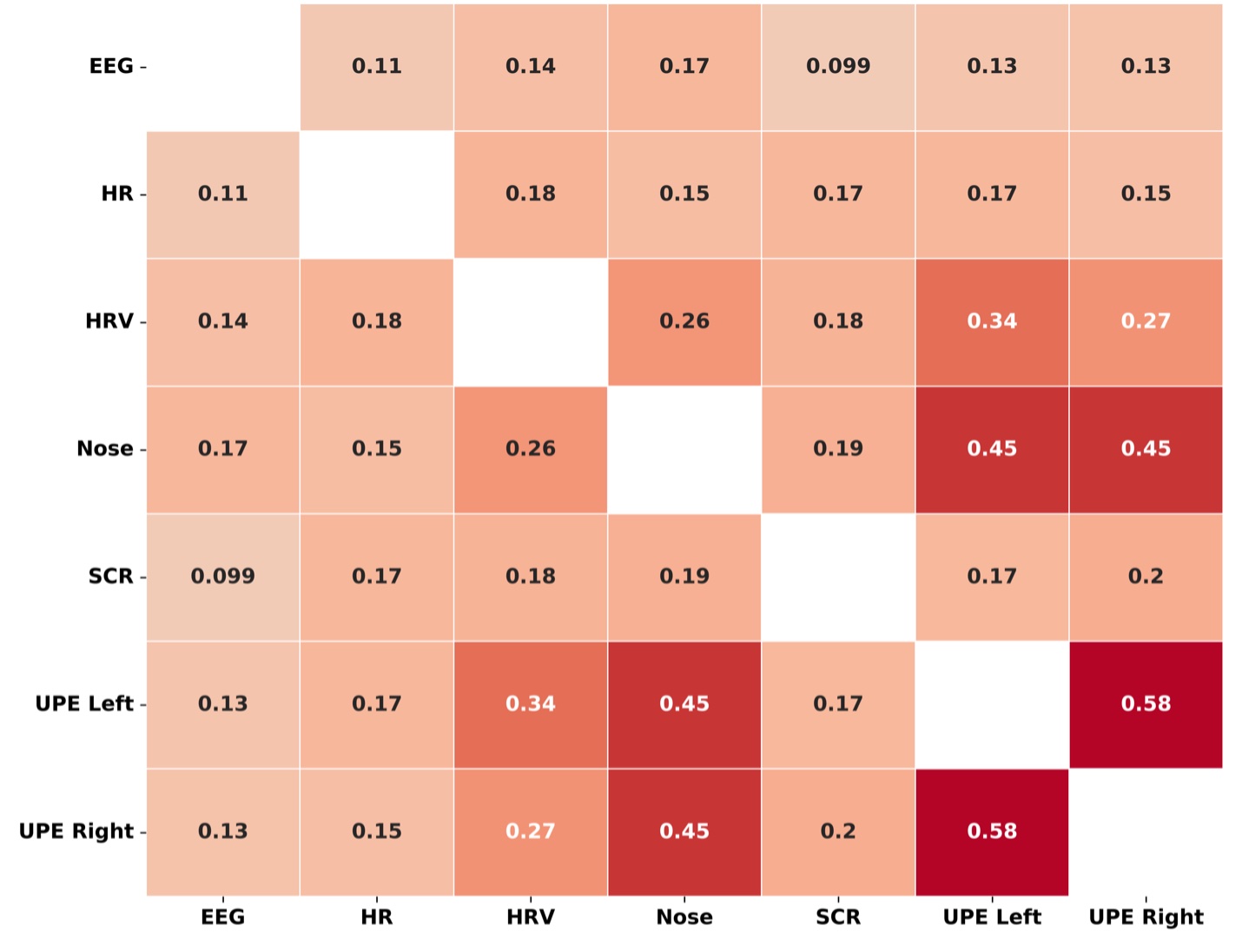

Supplement: SUPPLEMENTARY FIGURE 4 — Correlations (r) between absolute values of biofield measures from the meditation intervention after the Fisher z transformation. [file Image_4.jpeg]

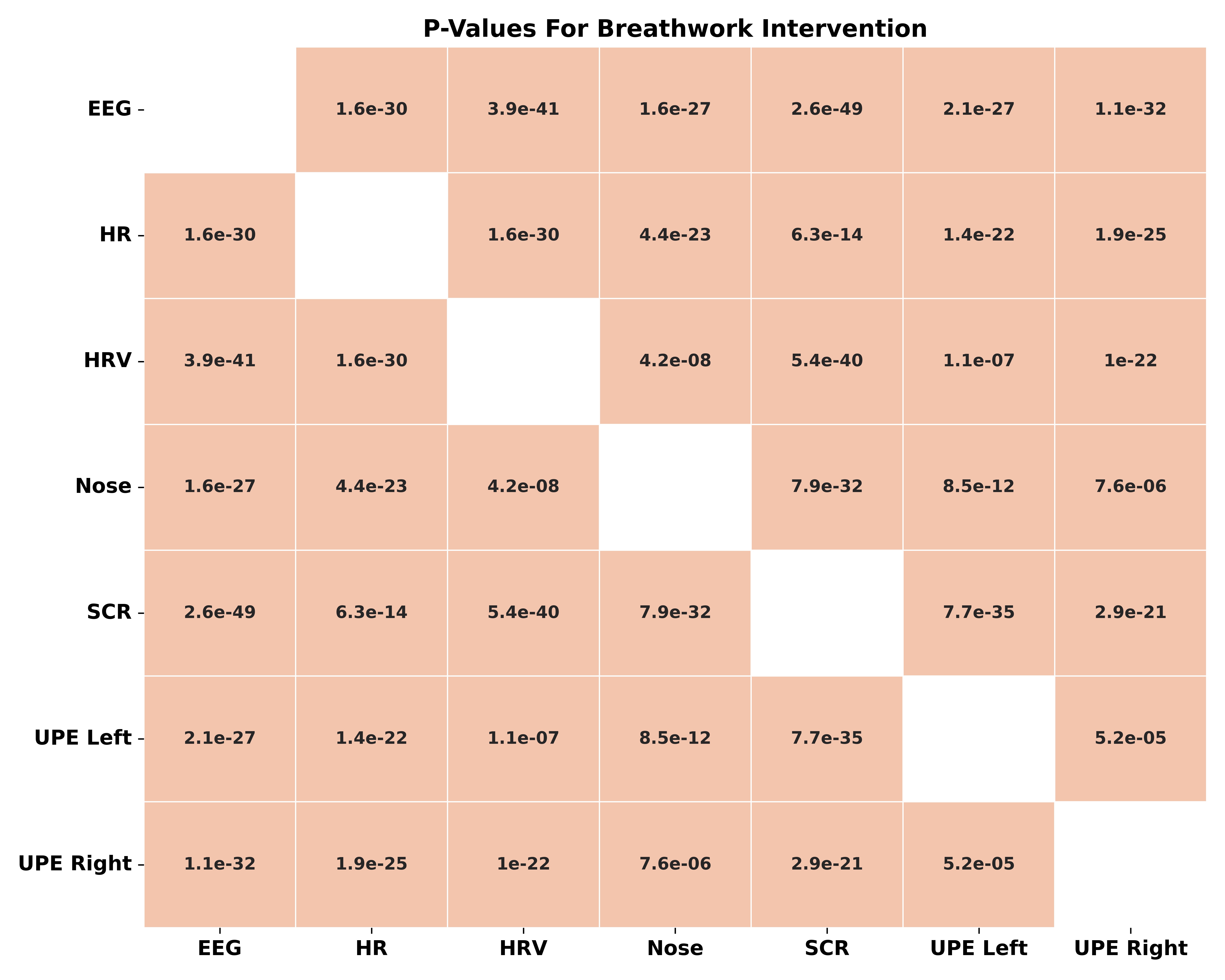

Supplement: SUPPLEMENTARY FIGURE 5 — P values from the correlational analyses for biofield measures from the breathwork intervention. [file Image_5.jpeg]

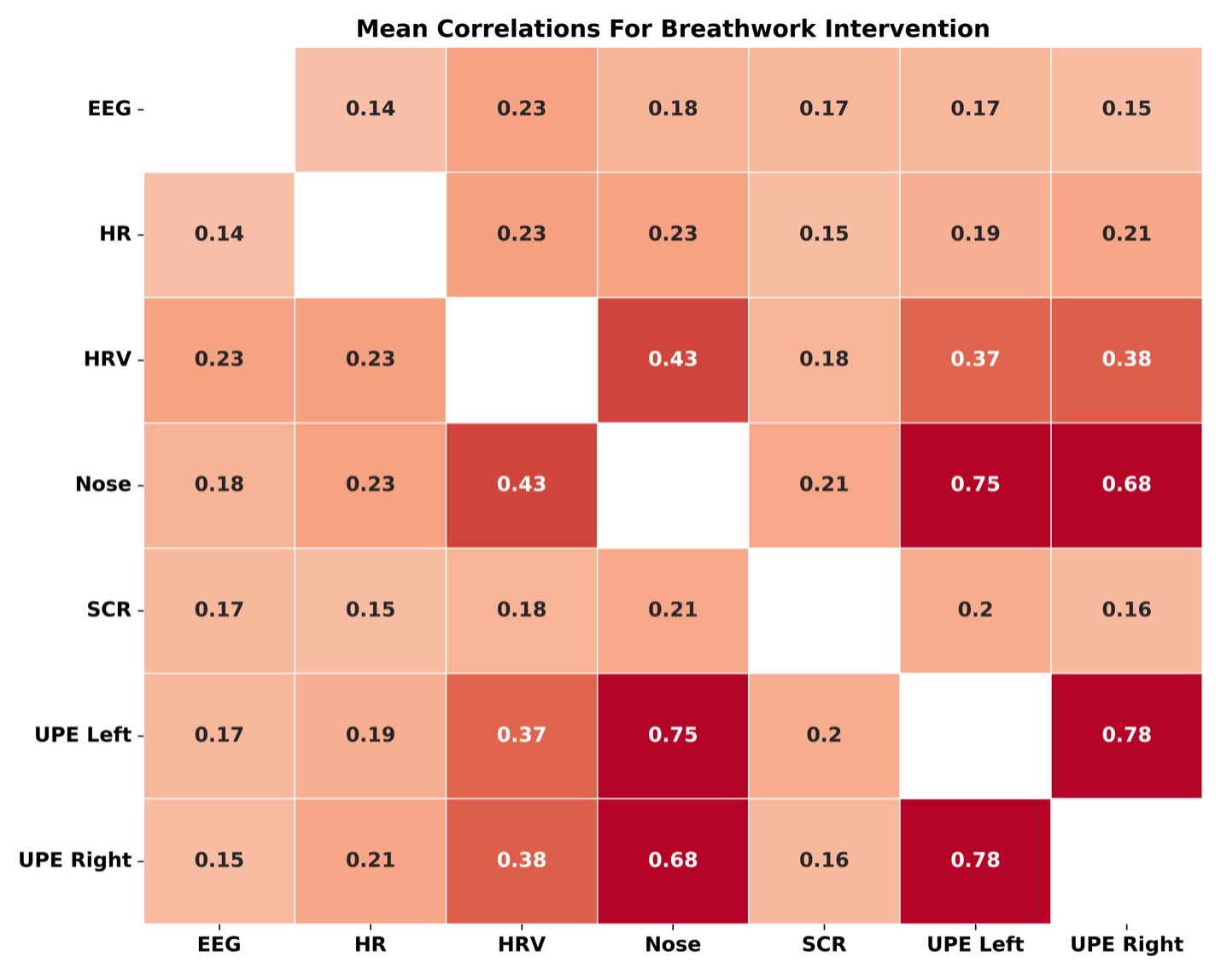

Supplement: SUPPLEMENTARY FIGURE 6 — Correlations (r) between absolute values of biofield measures from the breathwork intervention after the Fisher z transformation. [file Image_6.jpeg]
